# Transcriptomic profiling for identifying differentially expressed genes in aneurysm

**DOI:** 10.6026/973206300221789

**Published:** 2026-03-31

**Authors:** G. Shirley Lois, Shanmugapriya Murugan, Mohana Shanmugam Jaganathan, Dhanush Kumar S, Jino Blessy J

**Affiliations:** 1 Department of Bioinformatics, Sri Ramachandra Faculty of Engineering and Technology, Sri Ramachandra Institute of Higher Education and Research, Chennai, India

**Keywords:** Aneurysm, RNA sequencing (RNA-Seq), differential gene expression, protein-protein interactions (PPI), key genes

## Abstract

Aneurysm progression is associated with complex molecular alterations that are insufficiently studied at transcriptomics level. An aneurysm is characterized as a bulge or a weak spot in a blood artery's wall that causes the vessel to abnormally enlarge or balloon, exceeding 50% of its normal diameter. In the present study aneurysm RNA sequencing (RNA-Seq) dataset involving 14 samples, which include 7 controls and 7 treatments was selected for the analysis. Pathway analysis showed the involvement of key genes in major shifts within lipid metabolism pathways. The protein-protein interaction (PPI) network analysis using the STRING database identified key hub genes that were significantly differentially expressed, including LIPE, SREBF1, SCARB2, LPL, PNPLA2, UCP1, CIDEC, DGAT2, CIDEA and FABP4. These key gene-encoded proteins may be prominent drug targets for future interventions aimed at treating aneurysms.

## Background:

An aneurysm is a localized, abnormal bulge or dilation of a blood vessel caused by weakness in the vessel wall [1]. This anomaly can occur in both arteries and veins, the most commonly observed are cerebral, aortic and peripheral aneurysms [2]. This abnormal dilation can be due to a combination of various factors, including genetic, environmental and mechanical [3]. Intrinsic factors, such as age, sex, family history, hypertension, atherosclerosis, etc. and environmental conditions, smoking, diet and lifestyle choices contribute to the pathophysiology of aneurysm. These multifactorial mechanisms severely affect blood vessel health, causing weakness, which in turn leads to the bulging or ballooning of the vessel. In early stages, aneurysms are often asymptomatic and remain undiscovered until they reach a critical size or rupture [4]. For example, a cerebral aneurysm manifests with nonspecific symptoms like headache or dizziness, whereas aortic aneurysms present with minor or nonspecific symptoms like vague back and abdominal pain, which is chronic [5]. When these aneurysms rupture, they can result in fatal outcomes like severe headaches, neck stiffness, neurological deficits, photophobia or even seizures in case of cerebral aneurysms. For aortic aneurysms, sudden pain in the mid-abdomen, back, chest, varying degrees of shock and a pulsatile abdominal mass are observed [6]. The detection of aneurysms has progressively improved, given the advances in medical imaging procedures. These include non-invasive techniques like magnetic resonance imaging (MRI), computerized tomographic angiography (CTA), non-contrast computed tomography (CT) and ultrasound. Most of the unruptured aneurysms are identified when the patient gets imaging done for other reasons [7, 8]. In the case of high-risk individuals or in case of rupture, the above non-invasive procedures are done, but if they are negative and there is a sustained suspicion of rupture, invasive procedures for detection are performed. They include lumbar puncture (LP) and distal subtraction angiography (DSA) among other procedures [9].

Treatment strategies depend on various factors such as the location, size and risk of rupture of the aneurysms. Risk factor modification, like cessation of smoking, control of hypertension, hypercholesterolemia and regular moderate exercise, can help reduce the growth. In severe cases, surgical intervention options are considered, like endovascular aneurysm repair (EVAR), open surgical repair, microsurgical clipping and endovascular coiling [10]. Despite growing evidence linking metabolic dysregulation to aneurysm pathology, the transcriptomic alterations and key regulatory genes involved in this process remain insufficiently understood. To address this gap, the present study performed RNA-seq based differential expression and pathway analysis to characterize molecular changes associated with aneurysm. Using DESeq2, ShinyGO, STRING and Cytoscape, we identified altered pathways and key hub genes involved in lipid metabolism and energy homeostasis. Therefore, it is the internet to report transcriptomic alterations and to identify biomarkers and therapeutic targets associated with aneurysm progression.

## Materials and Methods:

## RNA sequencing analysis:

## Retrieval of aneurysm dataset and pre-processing:

Next-generation RNA sequencing samples were retrieved from the NCBI GEO Database, consisting of 14 samples, which include 7 controls and 7 treatments. The controls included: SRR27887013, SRR27887012, SRR19091709, SRR19091711, SRR24951143, SRR24951141 and SRR24951145 and the treatment samples included: SRR27887011, SRR27887010, SRR24951171, SRR19091704, SRR19091705, SRR24951153 and SRR24951152. The dataset was then imported into the Galaxy Server (https://usegalaxy.org.au/) using the "Faster Download and Extract Reads in FASTQ" tool (Galaxy Version 2.11.0 + galaxy0). The quality check of the reads was performed using the tool "FastQC" (Galaxy Version 0.73 + galaxy0). After which the tool "Trimmomatic" was used for sequence and adaptor trimming, making the data ready for further steps [11].

## Read alignment and differential expression functional analysis:

The sequence Mapping and Alignment were performed on the trimmed FastQ files using the tool "HISAT2", which is a fast and sensitive alignment program (Galaxy Version 2.2.1 +galaxy1). The annotations for gene regions were provided in GTF file format. The human genome reference version hg38 was used for this step. The output BAM files and annotation GTF file were analyzed using the tool "feature Counts" to measure gene expression in RNA-Seq data (Galaxy Version 2.0.1 + galaxy2). Differential expression gene analysis was performed on two groups, namely, Factor Control VS Factor Treatment, using the tool "DESeq2" (Galaxy Version 3.50.1+galaxy0) [12]. The gene set functional enrichment analysis was performed by ShinyGo, where a detailed gene ontology (GO) analysis was done and visualization of pathways, enrichments, gene characteristics and protein interactions were retrieved for further understanding and analysis [13].

## Protein-protein interaction network:

Protein-protein interaction network was built using the web-based database STRING. The top 25 differentially expressed upregulated genes with the parameters log2FC count ≥ 2.5 and p-value < 0.05 were selected from the DESeq results for this analysis [14]. Following the analysis, the interaction map was downloaded in TSV format for further visualization and analysis using Cytoscape. The relationship between genotypes, biological systems and gene expression was visualized using the Cytoscape software to find the hub genes with high confidence scores [15] and the CytoHubba plugin was used to retrieve the top 10 hub genes by ranking the nodes based on network topology.

## Results and Discussion:

Aneurysm DESeq analysis on a total of 14 samples, containing 7 control and 7 treatment, cluster analysis was done using principal component analysis plot, where there is 71% variance and 16% in PC2, indicating larger variance on the x-axis (PC1) (Figure 1 - see PDF). The hierarchical clustering based on the expression profiles of the sample was visualized using the heatmap (Figure 2 - see PDF). The heatmap reveals the similarity or distance between gene expression profiles of the aneurysm samples. In the heatmap, dark blue indicates a shorter distance and high similarity between samples and the lighter blues imply a higher distance and low similarity between the samples. There are two clusters seen, a small cluster at the top left and a larger cluster on the bottom right, with high similarities between the control samples and the same trend is seen with the treatment samples, indicating transcriptomic separation between the aneurysm dataset. The gene set enrichment analysis revealed that the aneurysm up and down regulated genes was involved in KEGG pathways (Figure 3 - see PDF).

The functional enrichment analysis of the genes that were performed using ShinyGo revealed the key lipid metabolism-related pathways, such as triglyceride catabolic process, cellular response to fatty acid, neutral lipid biosynthetic process, acylglycerol biosynthetic process, neural lipid catabolic process, lipid storage, triglyceride, acylglycerol and neutral lipid metabolic processes and lipid localization that are altered in the treatment group. These findings are supported by previous research linking disruptions in lipid metabolism to vascular pathology and aneurysm formation [2, 16]. Improper lipid metabolism may lead to abnormal accumulation of lipid and lipoproteins in the blood, which may gradually cause vascular accumulation and damage to vessels. The metabolic reprogramming affecting lipid storage and mobilization likely contributes to the vessel wall weakening and inflammatory processes in aneurysm pathophysiology [3]. The protein-protein interaction map that was derived from the CytoHubba plugin revealed the top 10 hub genes as: LIPE, SREBF1, SCARB2, LPL, PNPLA2, UCP1, CIDEC, DGAT2, CIDEA and FABP4 (Figure 4 - see PDF) and further literature research and protein function mapping confirmed that the proteins encoded by these hub genes are directly related to the regulatory pathways discussed above, validating the biological relevance of the enriched pathways in this study. This aligns with the research highlighting their involvement in vascular remodeling and energy homeostasis related to aneurysm disease [17, 18]. The identification of the key hub genes can serve as promising biomarkers or as drug targets to combat the development of aneurysms by modulating lipid metabolism.

## Conclusion:

We show important transcriptomic alterations related to aneurysm, with a strong emphasis on dysregulated lipid-metabolism pathways. The DEGs and identified hub genes involved in pathways like lipid storage, mobilization and vascular remodeling processes. These molecular insights reinforce the role of metabolic reprogramming in weakening vessel walls and contributing to aneurysm pathology. Our findings provide potential biomarker candidates and therapeutic drug targets, though further experimental validation is needed to confirm their functional relevance.

## Disclosure statement:

No potential conflict of interest was reported by the author(s).

## Funding:

No funding was received.

## Ethical approval:

Not applicable.

## Informed consent:

Not applicable.

## References

[1] Cho MJ (2023). Exp Mol Med..

[2] Gao J (2023). Signal Transduct Target Ther..

[3] Yang G (2024). MedComm..

[4] Golledge J (2023). Eur Heart J..

[5] https://www.ncbi.nlm.nih.gov/books/NBK507902/.

[6] https://www.ncbi.nlm.nih.gov/books/NBK470237/.

[7] Chung CY (2022). Radiographics..

[8] https://www.ncbi.nlm.nih.gov/books/NBK557867/.

[9] Liu J (2025). Sci Rep..

[10] Lin J (2023). Front Cardiovasc Med..

[11] The Galaxy Community. (2024). Nucleic Acids Res..

[12] Rosati D (2024). Comput Struct Biotechnol J..

[13] Ge SX (2020). Bioinformatics..

[14] Szklarczyk D (2021). Nucleic Acids Res..

[15] Shannon P (2003). Genome Res..

[16] Pan T (2023). Biomolecules..

[17] Zhang X (2019). Cells..

[18] Costa D (2023). Ann Vasc Surg..

